# Association between rural-to-urban migrants’ social medical insurance, social integration and their medical return in China: a nationally representative cross-sectional data analysis

**DOI:** 10.1186/s12889-019-6416-y

**Published:** 2019-01-18

**Authors:** Bo-li Peng, Li Ling

**Affiliations:** 1 Faculty of Medical Statistics and Epidemiology, School of Public Health, Sun Yat-sen University, Guangzhou, China; 2 Center for Migrant Health Policy, Sun Yat-sen University, Guangzhou, China

**Keywords:** Migration, Medical return, Social medical insurance, Social integration, China

## Abstract

**Background:**

Without social medical insurance in the destination areas and with low social integration, rural-to-urban migrants had barriers to health service in the destination areas, some of the migrants had to seek health service in hometown, namely medical return. This study aimed at exploring the association between rural-to-urban migrants’ medical return and social medical insurance type or social integration.

**Methods:**

We analysed a secondary cross-sectional data of the 2014 National Internal Migrant Dynamic Monitoring Survey collected in May of 2014 from all provinces or regions in mainland China. The medical return was measured by the location of hospitalisation, and the social integration included economic integration and permanent settlement intention.

**Results:**

Four thousand eighteen rural-to-urban migrants living in current residence at least one year and used inpatient service within the last 12 months were analysed. The rate of medical return for inpatient service was 15.3%. Having medical insurance of hometown (new rural cooperative medical scheme (NRCMS)) (OR = 2.44, 95%CIs 1.80–3.30) was positively related to the medical return. The permanent settlement intention was negatively associated with the medical return (OR = 0.66, 95%CIs 0.48–0.90).

**Conclusions:**

Social medical insurance of hometown (NRCMS) was positively associated with the medical return, while the permanent settlement intention was negatively associated with it. Promoting the transfer of migrants’ social medical insurance across different regions might be helpful to improve rural-to-urban migrants’ health access.

## Background

With the rapid economic development of the metropolis in China, the population of internal migrants has increased to 247 million in 2015, which accounted for 18% of the total population of China [1]. Internal migrants, including rural-to-urban migrants, were population living in their current residence over six months without a permanent/officially registered residence (*hukou*) of there [1]. The *hukou* system divided people into rural and urban residence. The rural-to-urban migrants accounted for 3/4 of the internal migrants in 2014 [2]. Lacking *hukou* in the destination areas, rural-to-urban migrants always have limited access to a range of social welfare provided by the local government, including housing, stable working, public health care services, and social medical insurance (SMI) [3–6].

The SMI system in China included the new rural cooperative medical scheme (NRCMS), urban resident-based basic medical insurance (URBMI) and urban employee-based basic medical insurance (UEBMI). URBMI was legal to internal migrants in few cities, 5.2% of migrants were enrolled in the URBMI of destination areas in 2014 [2]. Funded by employers and employees, UEBMI was friendly to the rural-to-urban migrants. In 2014, 23.6% of internal migrants were enrolled in the UEBMI of destination areas [2]. Most rural-to-urban migrants were enrolled in the NRCMS of hometown in 2014. Since the SMI was administrated by the local government, rural-to-urban migrants had much difficulty in transferring their SMI between different areas [7], thus suffered barriers in the reimbursement of their medical bill in destination areas in 2014. Even for the few cities which accepted the destination areas’ medical bill, rural-to-urban migrants always could receive low reimbursement; and the process was inconvenient and unpleasant [8]. Few cities (Shanghai, Chengdu, Shenzhen, Chongqing, etc.) had tried some special medical insurance for the rural-to-urban migrants from 2002, but the effect is limited. Most of these cities had terminated their special medical insurance before 2014 and continued to rely on the SMI system [2]. In other words, these rural-to-urban migrants enrolled in SMI of hometown always have to return for medical care to receive full reimbursement [7]. Someone has found that rural-to-urban migrants enrolled in UEBMI or URBMI were more likely to use inpatient services in their current residence compared with those enrolled in NRCMS of hometown [9].

Previous studies on rural-to-urban migrants’ returning home for health care had found that about 37.2% of 188 hospitalised migrants had returned (medical return) [10]. The main reasons for their medical return included the lower reimbursement for the medical cost in the host city, followed by high medical expenditure, and having nobody to take care of themselves [10].

Similar to rural-to-urban migrants, the medical return was also reported among international immigrant. Previous studies found that many Mexican immigrants living within 100 km of the U.S.-Mexico border had a medical return [11–13], as well as those living far away from the border [14]. The reasons for their medical return focused on the cost, medical insurance coverage, access, perceived medical quality, social integration, and preference on health service style [13–20]. Among these factors, medical insurance coverage and social integration were the most important factors. The social integration refers to the process of adapting to a new social environment [21]. Most studies showed a negative association between medical return and medical insurance coverage [13, 15, 16, 22, 23], social integration [24] and certain indicator of social integration, including language proficiency [15] and acculturation (measured by generation status) [25]. However, one study found no statistical significance between medical insurance coverage and medical return among Korean-U.S. immigrants. The explanation was that costs and social integration were more effective factors on medical return, and limited coverage of U.S. insurance on treatment would also push the immigrants away [26]. Correspondingly, many qualitative studies found that the maintenance of international immigrants’ original culture (another dimension of social integration [27]) would attract immigrants to return to seek health care. The reasons were as follows: feeling cultural comfort in homeland [15, 17, 18, 23], preferring the medical style of homeland [19], and having social connections [20] or social ties with homeland [23, 26].

Similarly, as the diversity of economic development across the rural and urban areas, rural-to-urban migrants in China also experience various level of social integration, including economic integration, cultural, social adaptation, social structural integration, and self-identity [28]. The economic integration was the fundamental of the social integration, which could be measured by employment status, household income, and housing. The self-identity was the final goal of social integration, which included the permanent settlement intention [27–29].

There has been plenty of research on international immigrants’ medical return, but rural-to-urban migrants’ medical return remains under-researched, and we have insufficient knowledge on the association between rural-to-urban migrants’ medical return and SMI or social integration. Although medical return could improve migrants’ access to health service, it also makes the service inconvenient and discontinuous. In this study, we applied the popular model of Anderson’s health behaviour model [30, 31] to analyse potential factors associated with the medical return, which was determined by the access to health service in different areas. Influencing factors in the model were divided into three categories, namely predisposing characteristics, enabling resources, and needs [31]. Some indexes of the three dimensions were also covered by the social integration. For instance, the enabling resources refer to the financial and social resources in hometown or destination areas, such as SMI and household income [20, 31], which also belong to the economic integration.

Based on the Chinese SMI system and previous studies on international immigrants’ medical return, we tested two hypotheses. (1) Rural-to-urban migrants enrolled in NRCMS would need to return in order to get full reimbursement, and thus would more likely to return for inpatient service compared with those enrolled in UEBMI or URBMI of current residence. (2)High social integration would be associated with good access to the social resource in current residence and being satisfied with the destination areas, and thus would attract migrants to use inpatient service at current residence.

Hence, we used data from the National Internal Migrant Dynamic Monitoring Survey (NIMDMS) in 2014 to assess the medical return (for inpatient service) of rural-to-urban migrants and to explore the association between the migrants’ medical return and their SMI type or social integration.

## Methods

### Data resource

This study performed a secondary analysis of the public access dataset NIMDMS [32], which was funded and organised by the National Population and Family Planning Commission of the People’s Republic of China (NPFPC) every year since 2009. The data in 2014 was selected because it was the latest NIMDMS collecting information on inpatient service utilisation of internal migrants, and information on the migrants’ social-demographic characteristics, social integration, and SMI status.

**Fig. 1:**
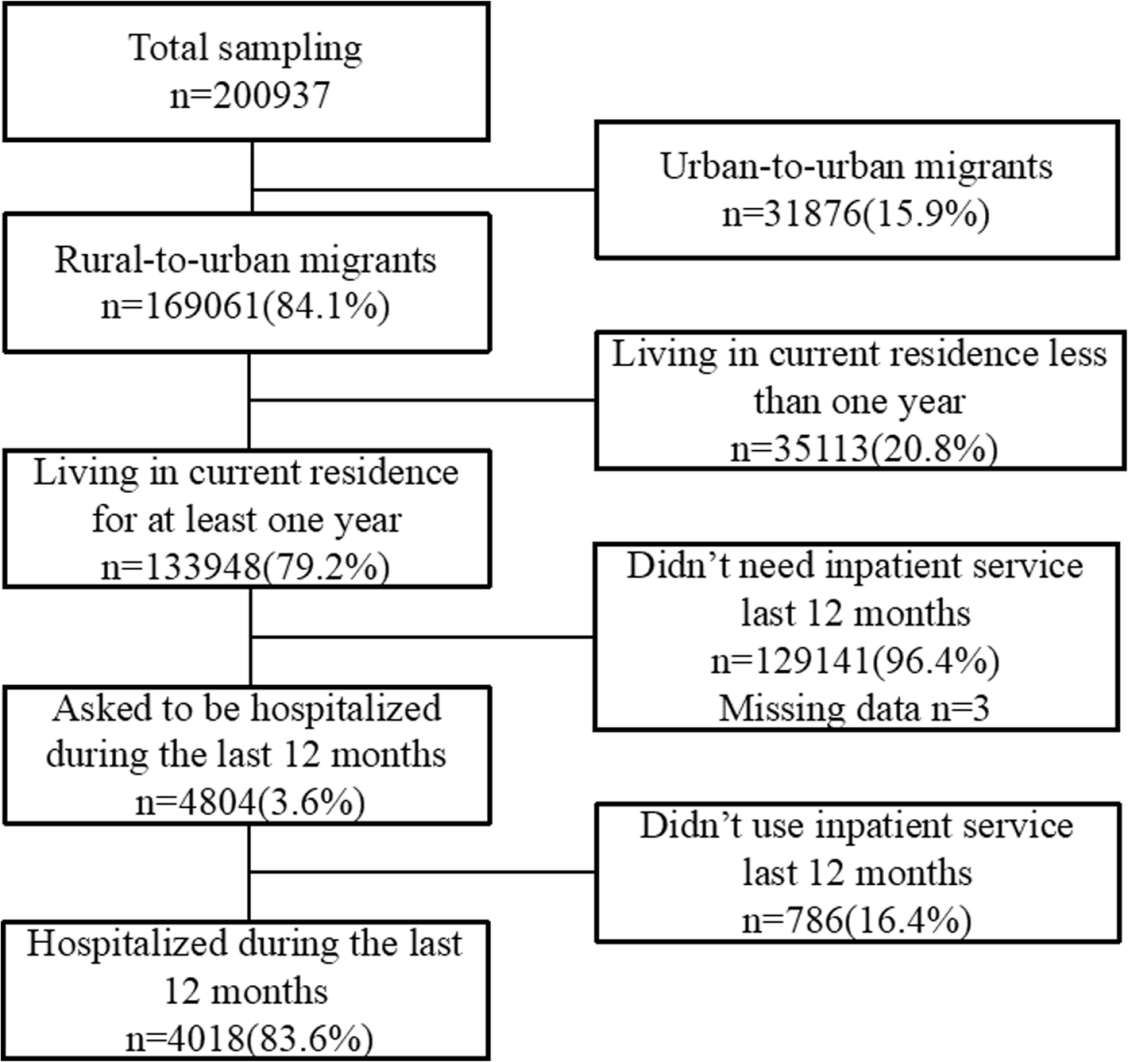
Flowchart of the sampling size of this study

The NIMDMS data of 2014 planned a nationally representative sampling of 201,000 internal migrants. The sampling was recruited from all 32 provinces and provincial regions in mainland China by a stratified, multi-stage, probability proportionate to size sampling method (PPS) in May of 2014. Details of the sampling process were presented in another literature [33]. The sampling framework of the NIMDMS was drawn from internal migrants’ size reported by the local government in 2013. Two-Level random sampling was conducted in strata (cities level) and townships to select the target townships, villages, and neighbourhoods. Twenty internal migrants aged 15 to 59 years old and lived in their current residence over one month were selected in each village or neighbourhoods. These internal migrants excluded students, and people for the purposes of training, tourism or health service [34, 35]. Investigators trained by the NPFPC and local health departments collected the data through the household survey. The quality control was conducted during the data collection and input. 200,937 internal migrants were recruited and completed the investigation in 2014, 169,061 (84.1%) of them were rural-to-urban migrants (Fig. 1).

### Participants

Participants in this study were limited to the rural-to-urban migrants who had been living in their current residence over 12 months before this survey and used inpatient service within that time. This inclusion criterion results in a sampling size of 4018 participants (Fig. 1). Rural-to-urban migrants were selected because they were the disadvantage but main subgroup of internal migrants. Additionally, the location of URBMI and UEBMI of urban-to-urban migrants could not be distinguished because of lacking this information in the NIMDMS. Besides, we only chose migrants living in their current residence for at least one year to exclude those migrants who used inpatient service in their hometown before their migration.

### Variables

#### Dependent variable

*Medical return* (measured by the location of hospitalisation).

The location of hospitalisation was categorised into current resident (=1), hometown (=2), and other places (=3); according to a self-reported question: where did you choose to be hospitalised last time during the last year? Medical return refers to the hospitalisation in hometown.

#### Independent variables

##### Social medical insurance

We collected the information on rural-to-urban migrants’ SMI status by asking them whether they were enrolled in NRCMS, URBMI, or UEBMI. Since the three types of SMI were administrated independently across rural and urban areas, rural-to-urban migrants could be enrolled in both NRCMS and URBMI or UEBMI. Hence, we divided the SMI status of rural-to-urban migrants into four categories:

1. Uninsured. Haven’t been enrolled in any SMI;
2. NRCMS of hometown. Rural-to-urban migrants were eligible to NRCMS of their hometown;
3. URBMI/ UEBMI of current residence. URBMI or UEBMI were eligible to rural-to-urban migrants in some destination areas;
4. Doubly insured. Migrants enrolled in both NRCMS and URBMI or UEBMI

##### Social integration

Referred to Zhou’s research on rural-to-urban migrants’ social integration [28], social integration can be divided into five dimensions, related to economic, cultural, society, structure, and self-identity. The social integration in this study was mainly drawn from the economic integration and self-identity. The economic integration act as the fundamental dimension of social integration and was measured by variables including employment status (0 = unemployed, 1 = employed), household income per member monthly (a continuous variable), and housing occupancy (1 = owned, 2 = rented, 3 = temporary shelter).

The permanent settlement intention, a critical index of self-identity, was measured by the willingness of staying in current cities in the future five years (0 = No, 1 = Unknown, 2 = Yes).

#### Confounding factors

The potential confounding factors were drawn from Anderson’s health behaviours model [30, 31]. The enabling resources were involved in the independent variables mentioned above.

Related to the predisposing factors, it contains:

1. Social-demographic characteristics: age groups, gender, ethnic group (1 = *Han* nationality, 2 = Minority ethnic), education level, marital status, and household size (1 = living alone, 2 = living with one family member (91.2% = 496/544 of them was spouse), 3 = living with two family members, 4 = living with three or more family members) in current residence; economic development of current residence (categorised into three levels according to the rank of consumption level per resident, 1 = developed province, 2 = developing province, 3 = under-developed provinces. Data source: China Statistic Yearbook 2015 Table 3–20 at http://www.stats.gov.cn/tjsj/ndsj/2015/indexch.htm).
2. Migration characteristics: migration type (migrated from other 1 = provinces, 2 = cities, and 3 = districts), the reason for migration (migrated for working or engaging in trade = 1, family reasons/visit relatives = 2, marriage = 3, other reasons = 4), and duration of staying in current residence (a continuous variable).

Needs were measured by a self-related question: why did you use inpatient service last time (1 = disease, 2 = injury or poisoned, 3 = delivery, 4 = other reasons).

### Data analysis

We used IBM SPSS (IBM crop, version 20.0) to conduct the data analysis. First, we described the rural-to-urban migrants’ demographic characteristics, medical return, social integration, and SMI status by the frequency distributions and percentages, means and standard deviations (SD). Second, we performed a chi-square test to analyse the relationships between medical return and the independent variables. Third, we carried out the multivariable multinomial logistic regression and stratified analysis to explore the association between medical return and SMI or social integration in the total rural-to-urban migrants and in different subgroups. Variables in the regressions were selected by the stepwise method under the threshold of *P* < 0.1. The unadjusted odds ratio (UOR), adjusted odds ratio (AOR), and 95% confidence intervals (CIs) were used to assess the association between medical return and independent variables. A two-side of *P* value less than 0.05 was considered statistically significant.

## Results

### Participants’ demographic characteristics

There were 4804 rural-to-urban migrants, 3.6% (=4804/133948) of the total rural-to-urban migrants, were asked to be hospitalised during the last 12 months before the survey. Among them, 4018 (83.6%) rural-to-urban migrants had used inpatient service (Fig. 1).

Table 1 showed the demographic characteristics of these rural-to-urban migrants. Most of them were female (2914, 72.5%), in the age group of 25 to 34 years (1964, 48.9%), married (3834, 95.4%), *Han* nationality (3616, 90.0%), living with two family members in current residence (1707, 42.5%), and haven’t reached the education level of high school (2760, 68.7%).

**Table 1:** Demographic characteristics of rural-to-urban migrants who used inpatient service

Variables	Rural-to-urban migrants hospitalised in			Total (*n* = 4018)	P value
	Current Residence (*n* = 3098)	Hometown (*n* = 614)	Other places (*n* = 306)		
Gender					< 0.001
Male	800(25.8)	181(29.5)	123(40.2)	1104(27.5)	
Female	2298(74.2)	433(70.5)	183(59.8)	2914(72.5)	
Age group					< 0.001
15-	566(18.3)	141(23.0)	33(10.8)	740(18.4)	
25-	1591(51.3)	269(43.8)	104(34.0)	1964(48.9)	
35-	610(19.7)	125(20.3)	98(32.0)	833(20.7)	
45–59	331(10.7)	79(12.9)	71(23.2)	481(12.0)	
Ethnic group					0.356
Han nationality	2786(89.9)	560(91.2)	270(88.2)	3616(90.0)	
Minority	312(10.1)	54(8.8)	36(11.8)	402(10.0)	
Education level					< 0.001
Never be educated	57(1.8)	11(1.8)	10(3.3)	78(1.9)	
Below high school	2011(64.9)	440(71.6)	231(75.5)	2682(66.8)	
High school	638(20.6)	103(16.8)	44(14.4)	785(19.5)	
College/Undergraduate/Postgraduate	392(12.7)	60(9.8)	21(6.8)	473(11.8)	
Marital status					0.257
Single	103(3.3)	20(3.2)	11(3.6)	134(3.3)	
Married	2959(95.5)	588(95.8)	287(93.8)	3834(95.4)	
Divorced/Windowed	36(1.2)	6(1.0)	8(2.6)	50(1.3)	
Household size					< 0.001
Alone	159(5.1)	47(7.7)	27(8.8)	233(5.8)	
With one family member	344(11.1)	138(22.5)	62(20.3)	544(13.5)	
With two family members	1354(43.7)	245(39.9)	108(35.3)	1707(42.5)	
With three or more family members	1241(40.1)	184(30.0)	109(35.6)	1534(38.2)	
Migrated from other					0.002
Provinces	1504(48.5)	292(47.6)	115(37.6)	1911(47.6)	
Cities	948(30.6)	198(32.2)	103(33.7)	1249(31.1)	
Districts	646(20.9)	124(20.2)	88(28.7)	858(21.3)	
Economic development of current resident					< 0.001
Developed	1366(44.1)	297(48.4)	71(23.2)	1734(43.2)	
Developing	839(27.1)	153(24.9)	140(45.8)	1132(28.2)	
Under-developed	893(28.8)	164(26.7)	95(31.0)	1152(28.7)	
Duration of stay in current residence	5.35 ± 4.63	4.94 ± 4.34	6.99 ± 5.96	5.41 ± 4.73	
Reason for migration					0.010
Working or engaging in trade	2251(72.7)	477(77.7)	248(81.0)	2976(74.1)	
Family reasons/visit relatives	740(23.9)	122(19.9)	48(15.7)	910(22.6)	
Marriage	81(2.6)	10(1.6)	8(2.6)	99(2.5)	
Other reasons	26(0.8)	5(0.8)	2(0.7)	33(0.8)	

In addition, most of these hospitalised migrants came from another province (1911, 47.6%), migrated for working or engaging in trade (2976, 74.1%), and lived in economically developed provinces of China (1734, 43.2%). These migrants stayed in their current residence for 5.4(SD = 4.7) years (Table 1).

### Rural-to-urban migrants’ SMI status and social integration

Among the 4018 hospitalised rural-to-urban migrants, 2776(69.1%) migrants were enrolled in the NRCMS, followed by UEBMI or URBMI (644, 16.0%), 200(5.0%) migrants were enrolled in both NRCMS and UEBMI or URBMI. However, there were 398 (9.9%) migrants have not been enrolled in any SMI (Table 2).

**Table 2:** Social integration and SMI status of rural-to-urban migrants who used inpatient service

Variables	Rural-to-urban migrants hospitalised in			Total (n = 4018)	P value
	Current Residence (n = 3098)	Hometown (n = 614)	Other places (n = 306)		
*Social integration*					
Employment					< 0.001
Yes	1770(57.1)	396(64.5)	204(66.7)	2370(59.0)	
No	1328(42.9)	218(35.5)	102(33.3)	1648(41.0)	
Household income per member (median, P_25_-P_75_)	1666.67 (1125.00–2500.00)	1666.67 (1200.00–2616.67)	1500.00 (1000.00–2500.00)	1666.67 (1125.00–2500.00)	
Housing Occupancy					< 0.001
Owned	809(26.1)	93(15.1)	119(38.9)	1021(25.4)	
Rented	2207(71.2)	499(81.3)	179(58.5)	2885(71.8)	
Temporary shelter	82(2.7)	22(3.6)	8(2.6)	112(2.8)	
Permanent settlement intention					< 0.001
Yes	2263(73.1)	365(59.4)	215(70.2)	2843(70.8)	
Still unknown	623(20.1)	183(29.8)	70(22.9)	876(21.8)	
No	212(6.8)	66(10.8)	21(6.9)	299(7.4)	
*Social medical insurance status*					< 0.001
None	335(10.8)	30(4.9)	33(10.8)	398(9.9)	
NRCMS	2069(66.8)	501(81.6)	206(67.3)	2776(69.1)	
NRCMS&URBMI/UEBMI	157(5.1)	25(4.1)	18(5.9)	200(5.0)	
URBMI/UEBMI	537(17.3)	58(9.4)	49(16.0)	644(16.0)	
The reason for being hospitalised					< 0.001
Disease	969(31.3)	230(37.5)	181(59.2)	1380(34.3)	
Injury	277(8.9)	30(4.9)	31(10.1)	338(8.4)	
Delivery	1704(55.0)	317(51.6)	68(22.2)	2089(52.0)	
Other reasons	148(4.8)	37(6.0)	26(8.5)	211(5.3)	

The social integration was measured by economic integration and the permanent settlement intention (a critical index of self-identity). Regarding the economic integration, 2370 (59.0%) out of 4018 migrants were employed. 2885 (71.8%) migrants lived in a rented house, compared with 1021 (25.4%) of migrants living in their own house in current residence. The median of the migrants’ household income per family member was 1666.7 (*p*_*25*_*-p*_*75*_: 1125 to 2500) *yuan RMB*. Regarding the permanent settlement intention, 2843(70.8%) migrants wanted to stay in their current residence in the future five years (Table 2).

### Rural-to-urban migrants’ medical return

Among 4018 rural-to-urban migrants used inpatient service, 3098 (77.1%) migrants were hospitalised in their current residence, 614 (15.3%) in their hometown, and 306(7.6%) in other places. They were hospitalised mainly for delivery (2089, 52.0%), followed by disease (1380, 34.3%) and injury or poison (338, 8.4%) (Table 2).

The migrants hospitalised in their current residence were different from those in hometown or other places on SMI status and social integration (Table 2).

### Association between medical return and SMI or social integration

After adjusting rural-to-urban migrants’ demographic characteristics(gender, age group and household size), migration characteristics(migration type) and the reason for hospitalisation and the economic development level of destination areas, results of the multivariable multinomial logistic regression indicated that medical return was positively associated with SMI in hometown and employed. Rural-to-urban migrants enrolled in the NRCMS of hometown preferred inpatient service in hometown (AOR of hometown vs residence = 2.44, 95%CIs 1.80 to 3.30) compared with those enrolled in URBMI/UEBMI of destination areas. Migrants employed also preferred hometown (AOR of hometown vs residence = 1.29, 95%CIs 1.05 to 1.60) compared with those unemployed. However, the permanent settlement intention was positively associated with being hospitalised in the current areas of residence (AOR of hometown vs residence =0.66, 95%CIs 0.48 to 0.90) (Table 3).

**Table 3:** Factors associated with rural-to-urban migrants’ medical return

Variables	Used inpatient service in ^a^			
	Hometown		Other places	
	UOR(95% CIs)	AOR(95% CIs)	UOR(95% CIs)	AOR(95% CIs)
Gender				
Male	1.20(0.99,1.45)	1.34(1.02,1.77)^*^	1.93(1.52,2.46)^***^	0.87(0.65,1.17)
Female(ref)	1	1	1	1
Age group				
15-	1.04(0.77,1.42)	0.99(0.68,1.43)	0.27(0.18,0.42)^***^	0.65(0.40,1.06)
25-	0.71(0.54,0.93)^*^	0.76(0.55,1.06)	0.30(0.22,0.42)^***^	0.76(0.52,1.11)
35-	0.86(0.63,1.17)	0.93(0.67,1.29)	0.75(0.54,1.05)	1.04(0.73,1.49)
45-(ref)	1	1		1
Household size				
Alone	1.99(1.39,2.86)^***^	2.08(1.40,3.09)^***^	1.93(1.23,3.04)^**^	1.44(0.88,2.37)
With one family member	2.71(2.11,3.48)^***^	2.62(1.99,3.45)^***^	2.05(1.47,2.87)^***^	1.46(1.02,2.10)^*^
With two family members	1.22(0.99,1.50)	1.23(0.99,1.52)	0.91(0.69,1.20)	1.02(0.76,1.36)
With three or more family members(ref)	1	1	1	1
Migrated from other				
Provinces(ref)	1	1	1	1
Cities	1.08(0.88,1.31)	1.37(1.10,1.71)^**^	1.42(1.08,1.88)^*^	1.09(0.80,1.49)
Districts	0.99(0.79,1.24)	1.16(0.90,1.51)	1.78(1.33,2.39)^***^	1.20(0.86,1.67)
Economic development of Current Resident				
Developed(ref)	1	1	1	1
Developing	0.84(0.68,1.04)	0.72(0.56,0.92)^**^	3.21(2.38,4.33)^***^	2.29(1.63,3.22)^***^
Under-developed	0.84(0.69,1.04)	0.73(0.58,0.92)^**^	2.05(1.49,2.82)^***^	1.79(1.27,2.52)^**^
Employment				
Yes	1.36(1.14,1.63)^**^	1.29(1.05,1.60)^*^	1.50(1.17,1.92)^**^	0.91(0.69,1.22)
No(ref)	1	1	1	1
Housing Occupancy				
Owned	0.43(0.26,0.72)^**^	0.66(0.38,1.15)	1.51(0.71,3.20)	1.88(0.86,4.08)
Rented	0.84(0.52,1.36)	0.96(0.58,1.58)	0.83(0.40,1.75)	1.06(0.49,2.27)
Temporary shelter (ref)	1	1	1	1
Permanent settlement intention				
Yes	0.52(0.38,0.70)^***^	0.66(0.48,0.90)^*^	0.96(0.60,1.53)	0.66(0.40,1.09)
No(ref)	1	1	1	1
Still unknown	0.94(0.68,1.30)	1.00(0.72,1.39)	1.13(0.68,1.89)	1.10(0.65,1.86)
Social medical insurance status				
None	0.83 (0.52,1.32)	0.95(0.59,1.53)	1.08(0.68,1.71)	1.32(0.81,2.15)
NRCMS	2.24(1.68,2.99)^***^	2.44(1.80,3.30)^***^	1.09(0.79,1.51)	1.10(0.77,1.56)
NRCMS&URBMI/UEBMI	1.47(0.89,2.43)	1.42(0.85,2.36)	1.26(0.71,2.22)	1.40(0.77,2.53)
URBMI/UEBMI(ref)	1	1	1	1
The reason for being hospitalised				
Disease(ref)	1	1	1	1
Injury	0.46(0.30,0.68)^***^	0.41(0.27,0.63)^***^	0.60(0.40,0.90)^*^	0.65(0.43,0.99)^*^
Delivery	0.78(0.65,0.95)^*^	1.31(0.97,1.77)	0.21(0.16,0.29)^***^	0.28(0.19,0.41)^***^
Other reasons	1.05(0.71,1.55)	1.16(0.78,1.75)	0.94(0.60,1.47)	1.12(0.71,1.78)

^*^:p < 0.05;

^**^:p < 0.01;

^***^p < 0.001

a. ref of Y was current residence

### Other factors associated with the medical return

Besides, migrants living alone (OR = 2.08, 95%CIs 1.40 to 3.09) or only with one family member (OR = 2.62, 95%CIs 1.99 to 3.45) tended to use inpatient service in hometown instead of the current areas of residence compared with those living with three or more family members together (Table 3).

### Association of medical return and the independent variables in different subgroups

Related to migrants hospitalised for delivery, the association between medical return and SMI or employment were positive and consistent with the main study. These migrants enrolled in both NRCMS and URBMI/UEBMI were also more likely to give birth back home compared with those only enrolled in URBMI/UEBMI. However, regarding migrants hospitalised for other reasons, the association between the medical return and employment did not reach the statistical significance, which suggested the employment only associated with the medical return for delivery (Table 4).

**Table 4:** Factors associated with rural-to-urban migrants’ medical return for different reasons

Variables	Hospitalised for delivery in ^a^ (*N* = 2089) AOR(95% CIs)		Hospitalised for disease/injury or other reasons in ^a^(*N* = 1929) AOR(95% CIs)	
	Hometown (*N* = 317)	Other places (*N* = 68)	Hometown (*N* = 297)	Other places (*N* = 238)
Gender				
Male	–	–	1.37(1.04,1.81)^*^	0.87(0.65,1.18)
Female(ref)	–	–	1	1
Age group				
15-	2.04(1.10,3.78)^*^	0.51(0.22,1.18)	0.78(0.48,1.28)	0.54(0.30,1.00)^*^
25-	1.55(0.86,2.80)	0.50(0.24,1.05)	0.66(0.46,0.96)^*^	0.80(0.54,1.19)
35-	Ref	Ref	1.02(0.72,1.42)	1.01(0.70,1.45)
45-(ref)			1	1
Household size				
Alone	1.84(0.75,4.50)	2.43(0.67,8.79)	2.17(1.35,3.50)^**^	1.33(0.77,2.32)
With one family member	6.92(4.31,11.12)^***^	4.43(1.69,11.62)^**^	1.67(1.17,2.39)^**^	1.25(0.84,1.86)
With two family members	1.28(0.97,1.69)	0.98(0.57,1.68)	1.16(0.83,1.62)	1.08(0.76,1.52)
With three or more family members(ref)	1	1	1	1
Migrated from other				
Provinces(ref)	1	1	1	1
Cities	1.67(1.23,2.27)^**^	0.86(0.47,1.57)	1.10(0.79,1.53)	1.17(0.81,1.67)
Districts	1.54(1.06,2.23)^*^	0.84(0.42,1.67)	0.88(0.60,1.27)	1.27(0.87,1.87)
Economic development of Current Resident				
Developed(ref)	1	1	1	1
Developing	0.67(0.47,0.95)^*^	2.42(1.25,4.71)^**^	0.79(0.55,1.12)	2.29(1.53,3.42)^***^
Under-developed	0.63(0.45,0.87)^**^	1.80(0.95,3.41)	0.87(0.62,1.22)	1.82(1.21,2.75)^**^
Employment				
Yes	1.49(1.14,1.94)^**^	1.03(0.61,1.74)	0.87(0.62,1.21)	0.82(0.58,1.15)
No(ref)			1	1
Housing Occupancy				
Owned	0.62(0.27,1.43)	1.42(0.30,6.81)	0.66(0.32,1.37)	2.05(0.83,5.06)
Rented	1.01(0.47,2.18)	0.95(0.21,4.23)	0.92(0.47,1.82)	1.10(0.45,2.66)
Temporary shelter (ref)	1	1	1	1
Permanent settlement intention				
Yes	0.72(0.46,1.13)	0.79(0.32,1.95)	0.64(0.40,1.01)	0.59(0.32,1.09)
No(ref)	1	1	1	1
Still unknown	1.18(0.74,1.87)	0.85(0.32,2.27)	0.88(0.53,1.45)	1.15(0.61,2.17)
Social medical insurance status				
None	0.88(0.45,1.70)	2.81(0.91,8.67)	1.16(0.58,2.33)	1.15(0.65,2.05)
NRCMS	2.16(1.39,3.35)^**^	2.27(0.85,6.04)	2.68(1.74,4.13)^***^	0.95(0.65,1.40)
NRCMS&URBMI/UEBMI	2.16(1.11,4.22)^*^	3.19(0.82,12.35)	0.76(0.32,1.82)	1.13(0.58,2.22)
URBMI/UEBMI(ref)	1	1	1	1
The reason for being hospitalised				
Disease(ref)	–	–	1	1
Injury	–	–	0.42(0.27,0.63)^***^	0.66(0.43,1.00)
Other reasons	–	–	1.22(0.81,1.83)	1.13(0.71,1.80)

^*^:p < 0.05;

^**^:p < 0.01;

^***^p < 0.001

a. ref of Y was current residence

Associations between medical return and SMI among migrants in different areas were also in line with the main study. The employment status was only significantly associated with the medical return of migrants in under-developed areas (Table 5).

**Table 5:** Stratified analysis on factors associated with rural-to-urban migrants’ medical return across different areas

Variables	Developed areas(*n* = 1734)		Developing areas(*n* = 1132)		Under-developed areas(*n* = 1152)	
	Used inpatient service in hometown ^a^ (*n* = 297, 17.1%)	Used inpatient service in other places ^a^ (*n* = 71, 4.1%)	Used inpatient service in hometown ^a^ (*n* = 153, 13.5%)	Used inpatient service in other places ^a^ (*n* = 140, 12.4%)	Used inpatient service in hometown ^a^ (*n* = 164, 14.2%)	Used inpatient service in other places ^a^ (*n* = 95, 8.2%)
Gender						
Male	1.45(0.94,2.24)	0.79(0.41,1.53)	1.14(0.70,1.87)	0.93(0.60,1.43)	1.37(0.80,2.35)	0.86(0.50,1.50)
Female(ref)	1	1	1	1	1	1
Age group						
15-	0.90(0.51,1.59)	2.37(0.66,8.50)	1.16(0.57,2.35)	0.55(0.27,1.12)	1.04(0.51,2.14)	0.57(0.22,1.46)
25-	0.65(0.39,1.08)	3.06(1.02,9.22)^*^	0.78(0.41,1.48)	0.53(0.30,0.94)^*^	1.04(0.55,2.00)	0.88(0.43,1.82)
35-	0.81(0.48,1.36)	3.42(1.13,10.39)^*^	1.17(0.64,2.12)	0.66(0.39,1.11)	0.92(0.48,1.76)	1.59(0.84,3.03)
45-(ref)	1	1	1	1	1	1
Household size						
Alone	2.43(1.24,4.74)^**^	1.50(0.55,4.08)	1.78(0.86,3.67)	1.40(0.66,2.99)	2.62(1.29,5.32)^**^	1.40(0.53,3.64)
With one family member	3.12(2.09,4.68)^***^	1.08(0.53,2.23)	2.21(1.28,3.81)^**^	1.85(1.05,3.26)^*^	2.54(1.45,4.45)^**^	1.19(0.59,2.43)
With two family members	1.53(1.11,2.10)^*^	0.66(0.36,1.20)	0.86(0.56,1.31)	1.32(0.83,2.09)	1.24(0.83,1.86)	1.03(0.63,1.69)
With three or more family members(ref)	1	1	1	1	1	1
Migrated from other						
Provinces(ref)	1	1	1	1	1	1
Cities	1.60(1.16,2.21)^**^	1.66(0.91,3.02)	1.66(1.03,2.68)^*^	1.17(0.71,1.93)	0.98(0.65,1.50)	0.66(0.38,1.15)
Districts	1.15(0.68,1.93)	2.24(1.03,4.89)^*^	1.28(0.78,2.09)	1.08(0.64,1.81)	1.06(0.68,1.66)	1.01(0.59,1.71)
Employment						
Yes	1.13(0.83,1.53)	0.93(0.52,1.68)	1.21(0.80,1.84)	0.90(0.58,1.39)	1.63(1.08,2.45)^*^	0.93(0.55,1.58)
No(ref)	1	1	1	1	1	1
Housing Occupancy						
Owned	0.60(0.23,1.60)	1.13(0.23,5.66)	0.49(0.22,1.11)	1.77(0.62,5.01)	1.12(0.34,3.73)	3.46(0.44,27.47)
Rented	1.13(0.47,2.75)	0.87(0.19,3.94)	0.58(0.28,1.23)	0.88(0.32,2.43)	1.56(0.50,4.82)	1.96(0.25,15.28)
Temporary shelter (ref)	1	1	1	1	1	1
Permanent settlement intention						
Yes	0.60(0.38,0.92)^*^	0.76(0.28,2.05)	1.06(0.50,2.24)	0.37(0.18,0.78)^**^	0.59(0.32,1.08)	1.28(0.43,3.77)
No(ref)	1	1	1	1	1	1
Still unknown	0.88(0.55,1.41)	1.65(0.60,4.56)	1.43(0.65,3.14)	0.65(0.29,1.43)	0.99(0.53,1.86)	1.66(0.53,5.19)
Social medical insurance status						
None	0.87(0.48,1.59)	1.28(0.52,3.16)	1.40(0.48,4.06)	1.66(0.78,3.55)	0.73(0.22,2.45)	0.88(0.31,2.51)
NRCMS	2.32(1.57,3.44)^***^	1.22(0.63,2.34)	3.06(1.46,6.41)^**^	1.07(0.61,1.87)	2.38(1.20,4.75)^*^	1.23(0.63,2.42)
NRCMS&URBMI/UEBMI	1.18(0.61,2.27)	0.82(0.26,2.60)	1.28(0.32,5.21)	1.90(0.71,5.04)	2.45(0.83,7.23)	2.45(0.80,7.44)
URBMI/UEBMI(ref)	1	1	1	1	1	1
The reason for being hospitalised						
Disease(ref)	1	1	1	1	1	1
Injury	0.31(0.16,0.61)^**^	0.71(0.30,1.72)	0.86(0.45,1.65)	0.47(0.24,0.93)^*^	0.17(0.05,0.59)^**^	1.02(0.50,2.09)
Delivery	1.46(0.93,2.30)	0.24(0.11,0.49)^***^	1.48(0.82,2.67)	0.28(0.15,0.52)^***^	1.04(0.58,1.85)	0.31(0.15,0.61)^**^
Other reasons	0.99(0.53,1.85)	1.58(0.74,3.41)	1.87(0.85,4.14)	1.24(0.56,2.77)	1.16(0.55,2.44)	0.68(0.25,1.83)

^*^:p < 0.05;

^**^:p < 0.01;

^***^p < 0.001

a. ref of Y was current residence

## Discussion

This study suggested a positive association between rural-to-urban migrants’ medical return and SMI in hometown (NRCMS), but a mixed association between medical return and social integration (measured by employment, household income, housing and the permanent settlement intention). The medical return was positively associated with employed status but negatively associated with the permanent settlement intention.

### Association between rural-to-urban migrants’ medical return and SMI status

Association between medical return and SMI of hometown (NRCMS) was positive, which was consistent with the previous studies on internal migrants [9, 10] and international immigrants [13, 15, 16, 22, 23]. This positive association could be explained as follows. Since SMI in China was coordinated and managed by the local government, people were enrolled in and reimbursed by SMI according to their *hukou* and the location of their SMI [7]. Although in few areas the NRCMS accepted the medical bills beyond their counties, it still had a cumbersome procedure to receive the reimbursement. The proportion of reimbursement beyond hometown was also lower than that in hometown [8]. Rural-to-urban migrants enrolled in NRCMS of hometown had to return to get more reimbursement through a convenient procedure [7].

Similarly, rural-to-urban migrants enrolled in UEBMI or URBMI of current residence could receive more reimbursement in the destination areas, and thus were in better access to the health service of the destination areas. This result was consistent with other studies in China [9, 36]. Some effective measures to improve migrants’ health access in the destination areas may as follows. First, the government might try to offer more stable works to the migrants and guarantee more employees can be enrolled in the UEBMI. Second, promoting the portability of the SMI across different areas would be effective. Third, some efforts may also be done to facilitate rural-to-urban migrants’ access to the URBMI of the destination areas.

Besides, rural-to-urban migrants who were enrolled in both insurance of current residence (URBMI/UEBMI) and hometown(NRCMS) preferred to give birth back home. The explanation would be that these migrants could receive reimbursement in both hometown and current residence, and migrants enrolled in NRCMS of hometown also felt more familiar with the health system of hometown and had more social ties with hometown.

The uninsured migrants showed similar preference on current residences’ inpatient service with those enrolled in UEBMI or URBMI of destination areas. The main reason would be as follows. First, due to the expensive medical costs in current residence but the extra costs of transports and times for returning, the total cost of hospitalisation between hometown and current residence would be extremely close. Second, the quality of inpatient service in developed destination areas would be better than that in hometown.

### Association between rural-to-urban migrants’ medical return and social integration

The association between social integration (including economic integration and the permanent settlement intention) and medical return was complex. The permanent settlement intention, measured by the willingness of staying in their current residence in the future [28, 29], was negatively associated with the medical return [24]. Since the permanent settlement intention was collected during the survey, it might not measure the permanent settlement intention before the health care utilisation very well and thus could not predict the causal relationship between these two variables. However, previous studies have found that high-integrated migrants were more familiar with the health system and the reimbursement policy of current residence, which could facilitate their hospitalisation in there [20]. The permanent settlement intention also indicated the preference on current residence including their health system, which would attract them to be hospitalised in there [15, 17–19, 23]. Despite this limitation, our results would inform the future prospective studies the possible association between these two variables. Besides, our results indicated the complex association between social integration and medical return.

Regarding the dimension of economic integration (measured by employment, household income, and housing), it demonstrated a complex result. Being employed was positively associated with medical return compared with the unemployed [10], especially among migrants who were hospitalised for delivery. However, employment status didn’t associate with the medical return for other reasons. The reason might be that the employment status in the survey was categorised into employed and unemployed. We had limited information about the migrants’ working time, such as the migrants’ working type (full-time or part-time job), which was also an important variable associated with the migrants’ access to health service and economic status. Further study is needed to explore the effect of employment status on rural-to-urban migrants’ medical return more precisely.

Migrants’ household income and housing occupancy did not achieve the statistical significance after adjusting other factors. The former was consistent with previous studies [15, 18]. The reason might be that rural-to-urban migrants’ medical return was mainly determined by the SMI.

### Other factors associated with rural-to-urban migrants’ medical return

As a social resource, the household size was also associated with the medical return. Rural-to-urban migrants living with two family members or less preferred hometown’s inpatient service compared with those living with three or more family members together. This result was consistent with other studies on rural-to-urban migrants [10, 37, 38] and studies on the general population [39]. Family members living together would enable the migrants to be hospitalised in current residence by taking care of the sick person during their hospitalisation [10]. However, fewer family members living together means less support as more family numbers left behind [28, 29], thus more likely to seek care closer to the family back home [20, 23, 26].

### Limitation

There were several limitations of this study. First, the cross-sectional study cannot predict the causal relationship between variables, but it can inform the future intervention study to improve the convenience of health service among migrants. Second, lacking information on language proficiency and social connection, we only checked some indexes of social integration. It could not reflect the social integration comprehensively; despite which, we found a mixed association between medical return and indexes of social integration. Third, we failed to get information on health service satisfaction with inpatient service in different locations and the seriousness of the disease, which were also associated with the medical return. However, we detected the impact of the permanent settlement intention, which could also indicate satisfaction with current residence, including health service in there. Fourth, the rural-to-urban migrants in this survey only included those returned back to be hospitalised and came back to the destination areas during the 12 months before the survey, thus excluded those who returned back but haven’t come back to the destination areas yet before the survey. Finally, the high proportion of delivery among the rural-to-urban migrants in this survey indicated there might be selection bias during the sampling. We performed a stratified analysis to test the associations between medical return and independent variables in different subgroups of the reason for hospitalisation.

## Conclusion

This study indicated a positive association between SMI of hometown and medical return. This positive association suggested that the government might improve migrants’ health access through facilitating the transfer of SMI across different regions or increase rural-to-urban migrants’ access to local SMI and improve the proportion of reimbursement of SMI. Besides, we found the association between social integration and medical return was complex. The permanent settlement intention was negatively associated with the medical return. More prospective studies are needed to test the causal relationship between the permanent settlement intention and medical return in the future.

## Abbreviations

AOR: adjusted odds ratio
CIs: confidence intervals
NIMDMS: the National Internal Migrant Dynamic Monitoring Survey
NPFPC: the National Population and Family Planning Commission of the People’s Republic of China
NRCMS: new rural cooperative medical scheme
PPS: probability proportionate to size sampling method
SD: standard deviations
SMI: social medical insurance
UEBMI: urban employee-based basic medical insurance
UOR: unadjusted odds ratio
URBMI: urban resident-based basic medical insurance
